# Parasitic isopods in fish aquaculture: A review of current knowledge and future perspectives

**DOI:** 10.1007/s00436-026-08685-3

**Published:** 2026-05-14

**Authors:** Kerry A. Hadfield

**Affiliations:** Water Research Group, Unit for Environmental Sciences and Management, North-West University, Potchefstroom, South Africa

**Keywords:** Cymothoidae, Gnathiidae, Control, Treatment, Effects, Drivers

## Abstract

Aquaculture has become a dominant contributor to global seafood production, providing essential protein and supporting livelihoods while alleviating pressure on wild fish stocks. However, cultured fish face numerous health challenges, including infestations by parasitic isopods, notably cymothoid and gnathiid species, which cause significant economic losses through reduced growth, compromised health, and mortality. Infestations are usually influenced by host factors, environmental conditions, parasite traits, and aquaculture management practices, including cage design, stocking density, water quality, and interactions with wild fish populations. Pathological effects range from tissue damage and anaemia to impaired growth, secondary infections, and mortality, particularly in juvenile fish. Effective management relies on integrated strategies combining preventive husbandry, environmental manipulation, biological control, selective breeding, and targeted chemical interventions, with an emphasis on reducing reinfection and limiting resistance development. Emerging approaches, including immunotherapy, gene editing, natural compounds, nanotechnology, and biosensors, show promise for sustainable control but remain largely experimental and require further validation. Future research should focus on life-cycle dynamics, host–parasite interactions, and eco-friendly interventions to improve prevention and management in intensive aquaculture systems. This review synthesises current knowledge on parasitic isopods in aquaculture, highlighting the need for integrated, ecologically informed strategies to safeguard fish health, ensure production sustainability, and mitigate economic losses.

## Introduction

Aquaculture is one of the fastest-growing segments of global seafood production, playing a crucial role in meeting the world’s rising demand for aquatic foods. In 2022, farmed seafood production reached 130.9 million tonnes, surpassing capture fisheries for the first time and accounting for over 50% of total aquatic animal production globally (FAO, 2024). By 2025, total seafood production was expected to reach approximately 197 million tonnes, with aquaculture driving most of this growth (Baird Maritime, 2025). Projections indicate that aquaculture will continue to expand faster than wild capture, potentially accounting for 56% of global production by 2034 (OECD-FAO, 2025). Beyond production, this sector provides affordable animal protein, supports livelihoods, sustains global seafood supply, and reduces pressure on wild fish stocks.

Cultured fish are challenged by a combination of factors, including water quality, bacteria, viruses, and parasites (Ogawa 2005; Irshath et al. 2023; Timi and Buchmann 2023). Parasitic isopods, particularly cymothoid and gnathiid species, are known to affect fish aquaculture facilities worldwide, causing significant economic losses through fish mortality, reduced growth, fertility issues, and decreased marketable yield. Several studies have investigated isopods in aquaculture, including the work by Horton and Okamura (2001), as well as a more recent book chapter summarising the impacts of parasitic crustaceans on commercially important fish and shellfish (Nowak et al. 2020). Despite the accumulation of studies, effective management is still constrained by limited ecological knowledge of these parasites.

Within the order Isopoda, the superfamily Cymothooidea comprises eight families associated with fish: Aegidae White, 1850; Anuropidae Stebbing, 1893; Barybrotidae Hansen, 1890; Cirolanidae Dana, 1852; Corallanidae Hansen, 1890; Cymothoidae Leach, 1814; Gnathiidae Leach, 1814, and Tridentellidae Bruce, 1984. These families vary in their degree of host association, ranging from permanent parasites to taxa with only temporary interactions with their fish hosts. Although some of these isopods are often referred to as micropredators in literature, they are treated here as temporary parasites, since they obtain nourishment directly from a living host for a limited period and cause harm without *immediately* killing it, thereby fulfilling the core definition of parasitism. Although host mortality may occur, particularly in juvenile or heavily infected fish, this represents a pathological outcome rather than a predatory strategy.

Gnathiid isopods are one example of temporary ectoparasites, with only the juvenile stages being parasitic and feeding on the blood or lymph of their hosts, while the adults are free-living and benthic (Hadfield et al. 2025). They are distinct from most other isopods in possessing only five pairs of pereopods, rather than the usual seven, and occur exclusively in marine environments. Gnathiid juveniles attach predominantly to the fins and gills of teleost and elasmobranch hosts. Species identification relies primarily on the morphology of the adult male, with molecular techniques increasingly used to assist in the identification of other life stages (Grutter et al. 2000). Two juvenile forms are recognised, the unfed zuphea and the fed praniza, which pass through three moulting stages before developing into adults (Hadfield et al. 2025). Although males and females are morphologically distinct, identification is currently based on adult male characters, making the identification of parasitic juvenile stages particularly challenging.

Of all the aforementioned isopod families, the Cymothoidae includes the largest number of genera and species described prior to 1900 and 1950, a pattern most likely attributable to their comparatively large body size (Smit et al. 2014). These large isopods parasitise a wide range of host species and may occur in the gills, buccal cavity, body cavity, or on the external surfaces of their host fish. The life cycles and ecological strategies of cymothoids vary considerably among taxa, and generalisations within the family are not recommended (Horton and Okamura 2003). Cymothoids exhibit a hermaphroditic reproductive strategy, transitioning from male to female as part of their development. Adult cymothoid parasites are permanently attached to an individual host, while a free-swimming immature stage (manca) is responsible for locating a suitable host. These isopods are typically encountered in pairs, consisting of a larger female (used for identification) and a significantly smaller male. While the low number of adults usually poses little threat to the host, the infective mancae move between hosts (optional intermediate hosts) in search of their final preferred host, causing damage during the process (Fogelman and Grutter 2008). For a detailed overview of cymothoid biology and the generalised life cycle, see Hadfield et al. (2025).

Cymothoids are permanent ectoparasites capable of infecting multiple host species, negatively affecting host health and growth, with impacts that are often amplified in aquaculture settings due to high host densities and confined environments. Although host specificity varies among species, mass infections can lead to growth retardation, weight loss, reduced condition factors, and, in severe cases, mortality. Recent studies (such as Nowak et al. 2020) indicate that these parasitic isopods cause substantial economic losses across marine, brackish, and freshwater aquaculture systems worldwide, with outcomes strongly influenced by host species and environmental conditions. Consequently, effective management depends largely on preventive strategies, such as environmental manipulation, selection of resistant host species, and biological control, rather than post-infection treatments. Horton and Okamura (2001) highlighted the potential for aquaculture studies to enhance understanding of cymothoid ecology and complement information obtained from wild-caught fish. This review, therefore, focuses primarily on cymothoid isopods and their documented impacts on aquaculture, with the aim of contributing to a clearer understanding and management of these parasites.

### Overview

Aquaculture has ancient origins, thought to originate with freshwater pond culture in China at least 8,000 years ago. Early fish-keeping also developed in Africa and Europe, with more organised systems emerging in the Roman Empire and later expanding through trout culture in the 18th century (Timi and Buchmann 2022). Despite this long history, modern large-scale and intensive aquaculture developed mainly over the past century, with Asia now dominating global production and regions such as Chile, North America, and Europe driving parasite-focused research in response to industrial mariculture (Timi and Buchmann 2023).

Parasites only became a major concern in aquaculture with the development of commercial production, when high stocking densities made the impacts of infections on farmed fish increasingly visible. The first recorded instance of a cymothoid isopod infecting fish in aquaculture was from the Diana Pond in Corsica (Mediterranean Sea) in 1981 (Bragoni et al. 1983, 1984). This isopod, *Nerocila orbignyi* (Guérin-Méneville, 1832), typically found on the external surfaces and fins of its fish hosts, was reported on cultured sea bass (*Dicentrarchus labrax* Linnaeus, 1758), where it caused high fish mortalities. Around the same period, Paperna and Overstreet (1981) reported a gnathiid infestation in the Red Sea, although the species was not identified, while a cirolanid, *Cirolana diminuta* Menzies, 1962, was causing severe losses in Californian nearshore caged fish (Stepien and Brusca 1985).

A few years later, *Ceratothoa gaudichaudii* (Milne Edwards, 1840) [now *species inquirendum*] was observed infecting Chilean salmon (*Salmo salar* Linnaeus, 1758) farms, leading to significant weight loss in heavily parasitised fish (Sievers et al. 1995, 1996). Other early reports included *Emetha audouini* (Milne Edwards, 1840) in Greek sea bass farms (Papapanagiotou et al. 1999) and *Ceratothoa oestroides* (Risso, 1827) in Croatian sea bass and sea bream farms in the same year (Šarušić 1999). Since then, numerous additional cases of cymothoid infections in aquaculture have been reported (see Table 1); however, the information provided varies considerably among studies, leaving important gaps in our understanding of these infestations. As shown in Table 1, available records are sporadic and likely underestimate the true frequency and extent of outbreaks. Some cases may not have been captured in this compilation, as they were not readily identifiable during literature searches, including those that are less accessible, published in non-English sources, or not clearly documented as aquaculture-related infestations. The underrepresentation of isopod infestation records may be further compounded by the fact that such parasites are often observed but not formally recorded, due to limited awareness or taxonomic expertise (Oliveira and Tavares-Dias 2025), particularly when no obvious harm to the host is evident.

**Table 1:** Reports of parasitic isopods in aquaculture, including host species, geographic locality, prevalence and cumulative mortality (where reported), as well as the estimated economic losses. Losses have been converted to 2025 values where applicable and are expressed in US dollars

Date	Fish species	Isopod species	Locality	Cumulative Mortality (%)	Prevalence (%)	Original Loss (US$)	2025-Adjusted Loss (US$)	Reference
1979–1980	*Girella punctata; Seriola quinqueradiata*	*Mothocya parvostis*	Japan (Nagasaki)					Bruce 1986
1981	*Moolgarda crenilabis* (as *Crenimugil crenilabis*); *Planiliza subviridis* (as *Mugil subviridis*)	*Gnathia* sp.	Gulf of Elat (Red Sea)					Paperna and Overstreet 1981
1981–1982	*Dicentrarchus labrax*	*Nerocila orbignyi*	France (Corsica)	7–18	10–90	US$ 16,284	US$ 47,200	Bragoni et al. 1983, 1984
1982–1983	*Heterostichus rostratus*	*Cirolana diminuta*	USA (California)					Stepien and Brusca 1985
1984	*Oncorhynchus kisutch*	Ceratothoa gaudichaudii*	Chile					Bravo 1988
1986	*Paralichthys olivaceus*	Gnathiidae	Japan					Yoshida 1988
1990	*Salmo salar*	Ceratothoa gaudichaudii*	Chile					Alvarado et al. 1990
1993–1994	*Salmo salar*	Ceratothoa gaudichaudii*	Chile		33–98.2	US$ 1,571 per 1,000 fish	US$ 3,250 per 1,000 fish	Sievers et al. 1996
1997	*Dicentrarchus labrax*	*Emetha audouini*	Greece	10.75		US$ 4,515	US$ 8,580	Papapanagiotou et al. 1999
1997–2011	*Dicentrarchus labrax*; *Sparus aurata*	*Gnathia maxillaris*	Spain					Hispano et al. 2013
1998–1999	*Oreochromis niloticus*	*Alitropus typus*	Thailand	50–100		US$ 234–468	US$ 450–900	Chinabut 2002
1999?	*Dicentrarchus labrax*; *Sparus aurata*	*Ceratothoa oestroides*	Croatia	10–20		US$ 1.30–1.35 per kg	US$ 1.65–1.72 per kg	Šarušić 1999
1999	*Dicentrarchus labrax*	*Ceratothoa oestroides*	Turkey (Çeşme)		1–68	US$ 112–191 per ton	US$ 203–347 per ton	Horton and Okamura 2001
2000	*Sparus aurata*	*Ceratothoa parallela*	Greece	50		US$ 1,001	US$ 1,850	Papapanagiotou and Trilles 2001
2002	*Dicentrarchus labrax*	*Ceratothoa oestroides*	Croatia (Adriatic Sea)	10	26–28			Mladineo 2002
2003	*Mystus gulio*	*Cymothoa indica*	India	100	100			Rajkumar et al. 2005a
2003?	*Salmo salar*	*Ceratothoa banksii* (as *Ceratothoa* cf. *imbricata*)	Australia (Tasmania)		1.2			Nowak et al. 2004
2004–2005	*Dicentrarchus labrax*; *Sparus aurata*	*Ceratothoa oestroides*	Croatia (Adriatic Sea)		30			Mladineo 2006
2005?	*Lates calcarifer*	*Cymothoa indica*	India	16.54				Rajkumar et al. 2005b
2005–2006	*Dicentrarchus labrax*; *Sparus aurata*	*Ceratothoa oestroides*	Greece		13.7–20			Vagianou et al. 2006
2006–2007	*Lates calcarifer*	*Cirolana fluviatilis*	India	45				Sanil et al. 2009
2007–2008	*Latris lineata*	*Ceratothoa banksii*	Australia (Tasmania)		1–67			Andrews et al. 2013
2008	*Dentex dentex*	*Anilocra physodes*	Turkey (Aegean Sea)		28.5			Trilles and Öktener 2009
2008	*Boops boops*; *Dicentrarchus labrax*; *Sparus aurata*	*Ceratothoa oestroides*	Croatia (Adriatic Sea)		20; 3.3; 13.3			Mladineo et al. 2009
2009	*Thunnus orientalis*	*Nerocila phaiopleura*	Japan (Shirahama)					Nagasawa and Shirakashi 2017
2009–2011	*Colossoma macropomum*	*Braga patagonica*	Brazil (Macapá)		2.5			Dias et al. 2015
2009–2012	*Salmo salar*	*Ceratothoa banksii; Nerocila orbignyi*	Australia (Tasmania)		1–100			González et al. 2019
2011–2012	*Dicentrarchus labrax*	*Nerocila orbignyi*	Egypt (Azbat El Borg)		2–4			Noor El-Deen et al. 2018
2013?	*Colossoma macropomum*	*Braga patagonica*	Brazil (Macapá)		30			Tavares-Dias et al. 2014
2014?	*Crassilabrus undulatus* (as *Cheilinus undulatus*); *Epinephelus fuscoguttatus*	*Caecognathia coralliophila*	Malaysia (Borneo Island)					Thing et al. 2015
2015	*Argyrosomus regius*	*Ceratothoa oestroides*	Croatia (Adriatic Sea)		5–19.4			Čolak et al. 2018
2016	*Arapaima gigas*	*Braga nasuta*	Brazil (Pará)		0.3			de Jesus et al. 2017
2017	*Pagrus major*	*Ceratothoa verrucosa*	Japan (Mie Prefecture)		0.01			Nagasawa and Tanaka 2017
2017	*Girella leonina*	*Nerocila japonica*	Japan (Shirahama)					Nagasawa et al. 2018
2018	*Argyrosomus regius*	*Livoneca redmanii*	Egypt		60–94			Fadel et al. 2020
2019	*Labeo catla* (as *Catla catla*); *Channa striata*; *Cyprinus carpio*; *Wallago attu*	*Alitropus typus*	India (Kerala)		68.2; 69.8; 62.5; 44.4			Suresh et al. 2022
2022	*Piaractus brachypomus*	*Braga patagonica*	Brazil (Macapá)		3.2			Oliveira and Tavares-Dias 2025
2022	*Chelon ramada*	*Nerocila bivittata*	Egypt (Ismailia)		26.5			Eissa et al. 2025
2022–2023	*Dicentrarchus labrax*	*Livoneca* cf. *redmanii*	Egypt		2			Arafat et al. 2026

* *species inquirendum*

? assumed date of infestation

There are several possible factors contributing to isopod parasite infestations in aquaculture. These include stocking density, stock quality, environmental conditions, parasite ecology, type and quantity of food, and feeding strategies. However, the most likely source of infection is contact with infected wild fish. Bragoni et al. (1984) observed that the prevalence of *N. orbignyi* reached 90% in wild mullets feeding around sea bass cages, making them the most probable source of sea bass infestations. Infected wild fish often congregate around fish farms, attracted by the abundance of food and possibly as protection from predators. This creates an ideal environment for parasite transmission, as juvenile isopods on these wild hosts are in close proximity to new potential hosts. Free-swimming mancae or zuphea actively seek a host on which to feed, making farms with dense fish populations particularly vulnerable to new infestations.

Host specificity among cymothoids varies by species. Under aquaculture conditions, however, a modified specificity may occur, where cymothoids attach to caged hosts they would not normally parasitise in the wild. For example, Papapanagiotou et al. (1999) reported *Emetha audouini*, primarily a parasite of Sparidae and Centracanthidae, on *D. labrax*, an unusual host for this species in Greece. Interestingly, adjacent cages containing sea bream (*Sparus aurata* Linnaeus, 1758), a seemingly more likely host, remained uninfected. Furthermore, Horton and Okamura (2001) observed all life stages of *C. oestroides* on Turkish sea bass (*D. labrax*), a host not naturally infected by this parasite in the wild. The presence of all developmental stages indicated that the parasite had successfully completed a full host shift rather than using the fish as an intermediate host.

### Pathological effects of isopods on host fish

Parasitic isopod infections can cause a range of pathological effects in their fish hosts under both natural and aquaculture conditions. These effects range from physiological and biochemical disturbances to mechanical tissue damage, reduced growth, impaired feeding, impacted organs, parasitic castration of the gonads, and even mortality (see Nowak et al. 2020 for more detailed effects). As fish farming has expanded globally, the consequences of isopod infestations have become increasingly apparent, with reports highlighting not only direct impacts such as gill and skin damage, anaemia, and secondary infections, but also indirect effects, including poor body condition, stunted growth, and increased susceptibility to environmental stressors and pathogens. The pathogenicity varies among parasite species and cannot be generalised across isopods. The impact depends on factors such as the parasite species, life stage, host, locality, fish health, and environmental conditions.

#### Cymothoidae

Cymothoid-infected fish in aquaculture often exhibit signs of irritation, such as erratic swimming, sluggish movement, biting on hard substrates in the cage (e.g., ropes or nets), and gaping mouths (Rajkumar et al. 2005a). Infected fish initially exhibit restlessness that progresses to lethargy, often accompanied by vigorous escape behaviours such as rapid swimming, leaping, thrashing, and rubbing against surfaces in an attempt to dislodge the parasites (Fadel et al. 2020). Muscle reddening and excessive mucus production are also commonly observed on the external surfaces or within the buccal cavity of infected fish (Rajkumar et al. 2005a). The secretion of mucus is recognised as a host response aimed at mitigating irritation caused by the isopod.

Other clinical signs associated with cymothoid infections include reduced condition factor, osmotic stress-related mortality (from freshwater dips), deep ulcerations, extensive haemorrhaging, ocular injuries, exposure of head musculature, and secondary infections (Papapanagiotou et al. 1999). Infective juvenile cymothoids can also cause haemorrhaging and necrosis of the gill lamellae (Papapanagiotou et al. 1999; Noor El-Deen et al. 2018). In Greece, fish infected with *Ceratothoa parallela* (Otto, 1828) displayed poor condition, lesions, and erratic swimming behaviour (Papapanagiotou and Trilles 2001). As a buccal-inhabiting isopod, *C. parallela* can settle on the tongue region, interfering with food intake and manipulation, which may lead to reduced feeding and emaciation.

A 2006 study reported that immature cymothoid stages caused extensive granulomatous lesions in the eyes of smaller sea bass, accompanied by an increase in inflammatory cells and haemorrhage, with total eyeball loss in severe cases (Vagianou et al. 2006). In addition, cymothoid-infected fish are often more susceptible to secondary infections and may act as vectors for pathogens, as bacteria and viruses have been detected in open lesions caused by the isopod’s attachment to the host (Vagianou et al. 2006; Rameshkumar et al. 2013). Recently, Eissa et al. (2025) linked *Nerocila bivittata* (Risso, 1816) to mass mortalities in farmed *Chelon ramada* in Egypt and suggested its role as a vector of *Photobacterium damselae* subsp. *piscicida*. The bacteria were detected both morphologically and molecularly within the isopod, indicating that infestations contribute to significant economic losses through increased mortality, reduced growth, and heightened host susceptibility to prolonged bacterial infections.

Bragoni et al. (1983) reported significant changes in the biochemical and haematological profiles of fish parasitised by cymothoid isopods. Infected fish exhibited reduced body condition and weight, as well as decreased levels of blood proteins, total lipids, triglycerides, and cholesterol, accompanied by elevated blood sugar, magnesium, phosphorus, and urea. The authors also observed hypochromic macrocytic anaemia, increased eosinophil and neutrophil counts, and decreased lymphocyte levels. Following a reduction in parasite load, fish showed marked improvement in body condition and biometric parameters, including increased blood proteins and lipids, decreased blood urea, and enhanced erythropoiesis (Bragoni et al. 1983).

Horton and Okamura (2003) confirmed that all life stages of *C. oestroides* are obligate blood feeders. In their study, parasitised fish exhibited significantly lower erythrocyte counts, haematocrit, and haemoglobin values compared to uninfected individuals, resulting in post-haemorrhagic anaemia due to blood loss. Conversely, leucocyte levels were elevated, with a relative increase in lymphocytes and granulocytic cells and a decrease in thrombocytes, suggesting an immune response to the parasite. Further studies are required to determine whether these haematological changes are reliable indicators of fish health (Horton and Okamura 2003).

The severity of pathological effects correlates with parasite load. Higher infestation intensity often leads to slower growth and reduced body weight in hosts (Lanzing and O’Connor 1975; Sievers et al. 1996; Horton and Okamura 2001; Čolak et al. 2018). Sievers et al. (1996) reported that Atlantic salmon (*Salmo salar*) infected with *Ceratothoa gaudichaudii* showed significant weight differences depending on the number of parasites: fish with fewer than three parasites averaged 4428 g, those with three to eight parasites averaged 4151 g, and fish with more than eight parasites averaged 3763 g. Fish with low parasite numbers (0–2 per host) appeared more tolerant and exhibited minimal changes in body mass. Similarly, Horton and Okamura (2001) found a 20% (14 g) reduction in mean weight and a 7% (13 mm) reduction in mean length in sea bass parasitised by *C. oestroides* compared to uninfected fish.

The condition factor of host fish is not always a reliable indicator of the impact of cymothoids. Williams and Bunkley-Williams (2000) found no significant difference in condition factor between parasitised and non-parasitised doctorfish, *Acanthurus chirurgus* (Bloch, 1787), although heavily parasitised fish were proportionally stunted. These same authors had previously reported that differences in condition factor in cero, *Scomberomorus regalis* (Bloch, 1793), only appeared when a female-male isopod pair occupied the gill cavity and had destroyed most gill filaments (Williams and Bunkley-Williams 1996). By considering fish age using otoliths, proportional stunting can be accurately assessed, revealing that parasitised fish are often lighter and shorter than uninfected individuals of the same age, circumventing the limitations of condition factor measurements.

Severe infestations have been noted to result in mortality, particularly in younger fish, often related to the release of infective isopod juveniles in search of a host. High numbers of juvenile isopods attaching to small fingerlings can damage gill filaments, impair respiratory function, and physically obstruct water flow through the buccal and gill cavities, leading to decreased feeding, anaemia, chronic emaciation, and ultimately mortality (Šarušić 1999; Horton and Okamura 2001; Mladineo 2002).

#### Gnathiidae

In gnathiid infestations, larvae often cluster on fish, causing areas of depigmentation or haemorrhage (Marino et al. 2004). Severe fusion and necrosis of secondary gill lamellae, along with subepithelial cellular exudation, occur near attachment sites. Large gnathiid infestations have caused 100% mortality in some studies, with fish hosting 100 praniza and 30 zuphea larvae in a single tank. Fish mortality was attributed to two main factors: mechanical damage to the skin, gills, and pharyngeal mucosa, and blood loss caused by the parasite’s feeding (haematophagy). Extensive gill infestation can lead to hypoxia due to loss of primary lamella function, while anaemia exacerbates respiratory failure. Individual gnathiids consume roughly 1.3–3.1 mg of blood, and high parasite densities induce severe anaemia, observable as pale organs at necropsy, ultimately causing death (Marino et al. 2004).

Jones and Grutter (2005) demonstrated that juvenile gnathiids significantly reduce haematocrit and total blood volume in captive wrasse, *Hemigymnus melapterus* (Bloch, 1791). While wild populations averaged 21 gnathiids per fish, captive fish were parasitised by 278–1251 gnathiids each. At these high infestation levels, some fish likely became anaemic, and continued exposure may have resulted in mortality as the gnathiid population increased during the study (Jones and Grutter 2005).

#### Effects of other isopods on host fish

*Alitropus typus* Milne Edwards, 1840, a well-known isopod from the family Aegidae, is recognised for causing significant losses in aquaculture. It infests a wide range of hosts and is a sanguivorous parasite, attaching to exposed body parts such as the mouth, gill chamber, and nostrils, or occasionally creating small pockets in the flesh of its host. Infested fish exhibit excess mucus production, abnormal swimming behaviour, scale loss, localised wounds, and reddening at attachment sites. Compared to non-infested fish, affected individuals appear weak, emaciated, and display pale gills (Suresh et al. 2022).

Within the family Cirolanidae, several species function as temporary parasites or micropredators. These isopods, in particular, blur the line between the two terms due to their destructive feeding behaviour. They are more aggressive than typical isopods, attacking fish and causing mass mortalities in a short period. In 1985, Stepien and Brusca noted that these isopods often entered fish through the anal opening, invading the body cavity and consuming gonads and liver, while musculature remained largely intact except in the most severe attacks. Moderate to heavy infestations also caused extensive gill damage, and isopods were sometimes found in the mouth and throat regions. According to a report by Sanil et al. (2009), *Cirolana fluviatilis* Stebbing, 1902, caused mass deaths of caged barramundi, *Lates calcarifer* (Bloch, 1790). Within the first two months, 35% of the fingerlings (8–12 cm) died, with many exhibiting severe tissue damage in which soft flesh was consumed, leaving only skeletal remnants. Isopods were observed feeding on moribund and dead fish, with 23–53 individuals per fish. Minor abrasions from nets or cages may have facilitated attachment. The fingerlings tested negative for Viral Nervous Necrosis (VNN), indicating that mortality was likely caused by isopod activity rather than a viral infection (Sanil et al. 2009).

### Impact of infestations by parasitic isopods

Isopod infestations can lead to significant economic losses. In Thailand, introduced red tilapia (*Oreochromis niloticus* Linnaeus, 1758) experienced 50–100% mortality within 2–7 days following infestation by *Alitropus typus*, most likely due to anaemia. This isopod, from the family Aegidae, occurs in the gills or buccal cavities of its hosts across tropical and subtropical parts of the Indo-Pacific. Under cultured conditions, 15–20 isopods can kill a small tilapia (5–8 cm) within six hours (Chinabut 2002). Given that tilapia is one of the most economically important cultured species, the financial loss per cage at the time (1999) was estimated at US$ 234–468 (based on a wholesale price of US$ 1–1.2/kg and 400–450 kg per cage) (Chinabut 2002). This loss would amount to approximately US$ 450–900 per cage in 2025.

In 2015, Shinn et al. (2015) reviewed the economic costs of parasites to mariculture, highlighting losses caused by five different isopods. *Ceratothoa gaudichaudii* infecting salmon (*Salmo salar*) in Chile (Sievers et al. 1996) was estimated to cause a loss of US$ 1,571 per 1,000 harvest-sized fish, equivalent to roughly US$ 3,250 per 1,000 fish in 2025. *Ceratothoa oestroides* on European seabass (*Dicentrarchus labrax*) at a Turkish site (Horton and Okamura 2001) resulted in losses of US$ 112–191 per kg, approximately US$ 203–347 per ton in 2025, and in Croatia caused a loss of US$ 1.4 per kg, updated to about US$ 1.7 per kg in 2025. This species also infested older sea bream (*Sparus aurata*) in Croatia (Šarušic 1999), causing a 20% reduction of growth, estimated at a loss of US$ 1.3 per kg, or about US$ 1.7 per kg in 2025. *Ceratothoa paralella* on *S. aurata* in Greece (Papapanagiotou and Trilles 2001) had an estimated value of US$ 3.9 per kg, corresponding to a loss of roughly US$ 1,850 in 2025. Although mortality exceeded 50% (over 120,000 juveniles), the relatively low biomass at this early life stage resulted in a comparatively small direct economic loss. However, the long-term production impact would likely have been higher if these fish had reached harvest size. The fourth species, *Emetha audouini*, reported from *Dicentrarchus labrax* in Greece, caused severe and deep skin damage to the cranial and ocular regions (Papapanagiotou et al. 1999), leading to estimated economic losses of US$ 0.7 per fish, or approximately US$ 8,580 per cage when adjusted to 2025 values. Finally, an infestation of *Nerocila orbignyi* in *Dicentrarchus labrax* in Corsica (Bragoni et al. 1984) resulted in an increase in mortality from 7% to 18% over a six-month period. At a value of US$11.2 per kg, this equates to an estimated economic loss of approximately US$47,200 in 2025 (see Table 1).

That said, the presence of isopods in aquaculture does not always lead to host mortality. For example, an outbreak of the gnathiid *Caecognathia coralliophila* in a fish hatchery on Borneo Island, Malaysia, not only did not affect the fish hosts, but also provided a unique opportunity to redescribe a poorly known gnathiid species (Thing et al. 2015). The gnathiid larvae, which fed on humphead wrasse (*Cheilinus undulatus* Rüppell, 1835) and tiger grouper (*Epinephelus fuscoguttatus* Forsskål, 1775), settled on dead coral rubble and rocks at the bottom of the enclosures, where they moulted into adults. The adult male, used for species identification, was confirmed as *C. coralliophila*, a species previously known only from a single male specimen. This outbreak allowed for a full redescription of the male, as well as the first descriptions of the female and larval stages.

### Drivers of infestation by isopods

**Fig. 1:**
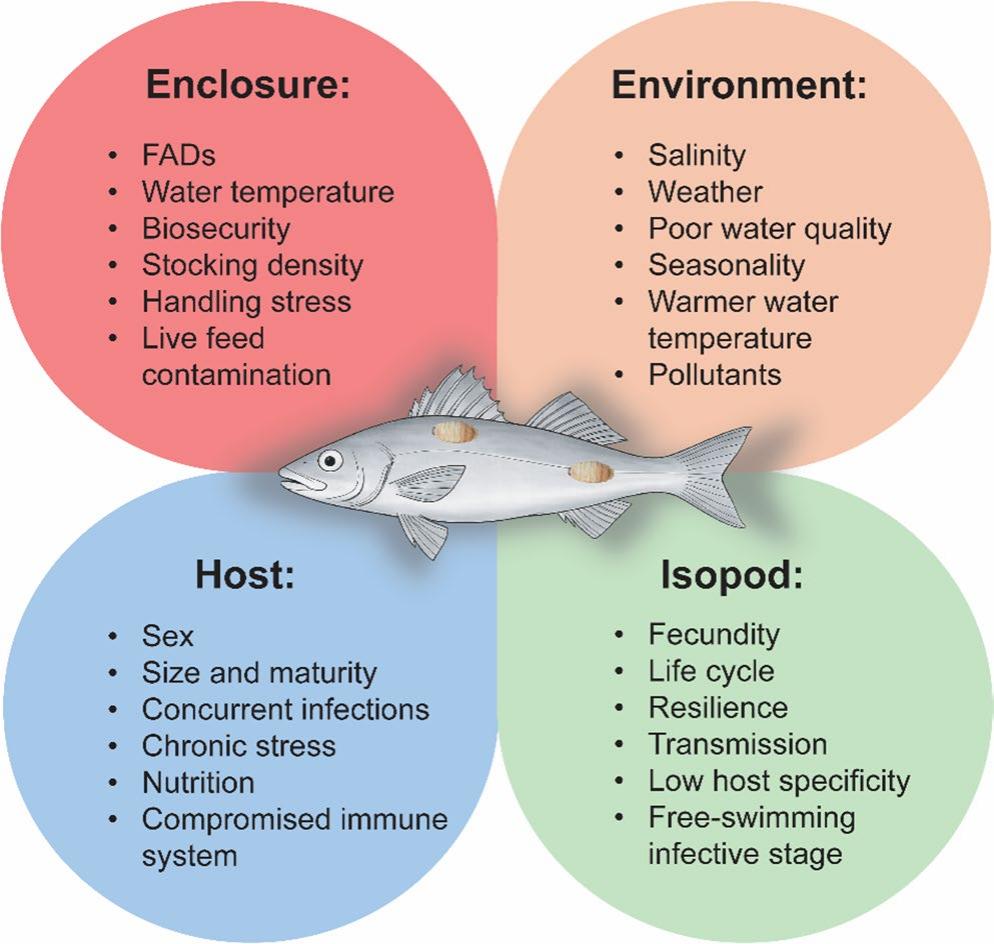
Conceptual model illustrating how environmental conditions, host susceptibility, parasite traits, and aquaculture enclosure facility factors interact to drive parasitic isopod infestations in fish aquaculture systems. Abbreviation: FADs = fish aggregation devices

Isopod infestations are influenced by a combination of parasite traits, host susceptibility, environmental conditions, and aquaculture management practices. Understanding these drivers is essential for developing effective prevention and control strategies. This section reviews the key factors contributing to isopod outbreaks, organised around parasite, host, environmental, and aquaculture/cage-related drivers (Fig. 1).

#### Enclosure management drivers

Aquaculture enclosures play a critical role in mediating parasite transmission by shaping host density, environmental conditions, and opportunities for parasite introduction. The cages act as fish aggregation devices (FADs), attracting large numbers of wild fish that persist around cages over time (Valle et al. 2007). These wild fish can act as parasite reservoirs, completing parasite life cycles and sustaining reinfection of farmed stocks.

Water temperature within enclosures strongly influences isopod development, reproduction, and survival. Elevated temperatures can accelerate parasite life cycles and increase the activity of free-swimming infective stages, while also potentially stressing fish and reducing their immune competence.

Biosecurity measures determine the likelihood of parasite introduction and spread. Poor biosecurity, such as inadequate screening of incoming stock, equipment sharing, or lack of quarantine procedures, allows isopods to enter and persist within systems, whereas strong biosecurity can effectively limit outbreaks.

Stocking density is a key amplifier of infestation risk. High densities increase physical contact among fish, facilitating parasite transmission and reducing the ability of hosts to avoid infective stages, while also contributing to stress and weakened immune responses (Yang et al. 2025). Stocking practices, cage design, and farm management are thus critical in controlling parasite prevalence.

Handling stress, associated with transport or routine management, compromises fish immune function and increases susceptibility to parasite attachment and establishment. Stressed fish are less capable of resisting infestation and may exhibit behaviours that increase exposure. Likewise, inadequate husbandry practices, such as poor removal of dead fish and limited monitoring, can perpetuate infestations, as observed with *Ceratothoa parallela* in cultured gilthead sea bream (*S. aurata*) (see Papapanagiotou and Trilles 2001).

Live feed contamination represents an important pathway for parasite introduction, particularly when wild-caught zooplankton are used. These feeds can act as vectors for infective isopod stages, enabling direct entry into aquaculture systems and initiating infestations. For example, *Cymothoa indica* infections in cultured Asian seabass (*Lates calcarifer*) were attributed to infective larvae introduced via wild-caught zooplankton (Rajkumar et al. 2005b).

#### Environmental drivers

Environmental conditions strongly influence parasite transmission and severity. Salinity affects parasite survival, reproduction, transmission, and host susceptibility (Yang et al. 2025). Many parasitic isopods have optimal salinity ranges, and stable or favourable salinity conditions can enhance their persistence and infectivity, while fluctuations may either limit or, in some cases, stress hosts and increase susceptibility.

Weather conditions (including storms, flooding, or monsoons) can disrupt aquaculture systems, damage cages, and introduce wild fish or parasite stages. These events can increase parasite transmission by enhancing water movement, redistributing infective stages, and facilitating contact between cultured and wild hosts.

Poor water quality, including low dissolved oxygen, high ammonia, or elevated organic loads, compromises fish health and immune function while promoting parasite survival and reproduction (Yang et al. 2025). Stressed fish are more vulnerable to parasite attachment and less capable of resisting infestation, while degraded conditions may also favour parasite survival in some cases.

Seasonality influences parasite abundance through cyclical changes in environmental conditions such as temperature, host availability, and reproductive timing. Peaks in isopod infestations are often associated with specific seasons that favour rapid development and increased transmission.

Warmer water temperature accelerates isopod metabolism, development, and reproduction, often shortening life cycles and increasing the abundance of infective stages. At the same time, elevated temperatures can stress fish, further increasing their susceptibility to infestation. For example, in Croatia, warmer waters increased *C. oestroides* infestations on sea bass (*D. labrax*) and sea bream (*S. aurata*) (see Šarušić 1999). Persistent warm temperatures with minimal seasonal variation can support continuous reproductive activity in parasites like *Livoneca* sp. (Colorni et al. 1997).

Pollutants, including chemical contaminants and agricultural runoff, can weaken fish host immune systems and alter aquatic ecosystems. Sublethal exposure may increase host susceptibility while also indirectly affecting parasite dynamics by modifying environmental conditions.

#### Host drivers

Host-related factors strongly influence susceptibility to parasitic isopod infestation by determining the likelihood of parasite attachment, establishment, and persistence. Sex if the host can affect infestation patterns through differences in behaviour, physiology, or hormonal regulation, which may influence exposure and immune responses.

Size and maturity (age) are important, as larger or more mature fish often provide greater surface area and more stable attachment sites, although juveniles may be more vulnerable due to underdeveloped immune systems. Younger fish are particularly vulnerable to gill damage caused by juvenile isopods, leading to impaired respiration and mortality of 10–20%, whereas older fish primarily experience growth retardation (Šarušić 1999).

Concurrent infections can weaken host defences and create synergistic effects that increase susceptibility to isopod infestation.

Chronic stress, often resulting from environmental or management pressures (such as those mentioned above), suppresses immune function and reduces the host’s ability to resist or recover from parasitic attachment. Stressors such as handling, grading, transport, high stocking densities, nutritional deficiencies, and co-infections elevate stress hormones, suppressing immune responses and increasing vulnerability to infestation (Yang et al. 2025).

Nutrition plays a key role in maintaining immune competence, and deficiencies can impair resistance to parasites.

Ultimately, a compromised immune system, whether due to stress, poor nutrition, or disease, is a central driver that facilitates successful infestation and increases parasite burden in aquaculture systems.

#### Isopod drivers

Parasite-specific traits are central to determining infestation pressure and the success of parasitic isopods in aquaculture systems. Fecundity influences the number of offspring produced, with higher reproductive output increasing the abundance of infective stages in the environment. Life cycle traits, such as the high fecundity of ovigerous females (*C. oestroides* releases 450–550 juveniles per brood), contribute to mass infestations and high mortality rates (10–20%) in younger fish (Šarušić 1999). Similarly, *Livoneca* sp. can reproduce continuously in persistently warm waters, allowing year-round infestation (Colorni et al. 1997).

The life cycle of isopods, particularly the presence of direct development and multiple parasitic stages (Hadfield et al. 2025), facilitates rapid population build-up under favourable conditions. Direct life cycles allow completion of development entirely within aquaculture systems.

Parasitic isopods exhibit high resilience to environmental fluctuations, tolerating wide ranges in temperature, salinity, and oxygen levels, which enhances their survival and persistence within aquaculture systems. Parasites primarily enter aquaculture systems through environmental pathways, including contaminated water, carrier organisms, and pollution sources, enabling rapid dissemination through ecological networks.

Efficient transmission strategies, including direct host-to-host transfer (from fish in close proximity) and environmental dispersal (via water currents, fish migration, etc.), further promote infestation spread.

Low host specificity allows many isopod species to exploit a wide range of hosts, increasing their ability to establish in diverse aquaculture settings. The infective free-swimming manca can attach to multiple hosts before finding the final host (Fogelman and Grutter 2008).

Additionally, the presence of a free-swimming infective stage enhances dispersal and host-finding efficiency, enabling parasites to readily locate and infest cultured fish.

### Control of parasites on host fish

As aquaculture continues to expand, effective parasite control has become increasingly important, since intensification of production systems promotes parasite proliferation. In 2022, Buchmann provided a comprehensive review of control strategies for isopods in aquaculture, highlighting the need for integrated approaches. These include chemotherapeutants and biocides, medicinal treatments, as well as mechanical, biological, immunological, and genetic methods (through selective breeding) (Buchmann 2022). Alternating between chemical, medical, biological, and mechanical strategies may help delay the development of resistance to one method.

Despite available interventions, control of cymothoid infestations in aquaculture remains difficult. Čolak et al. (2019) reported that chemical treatment of sea bass infected with *C. oestroides* reduced prevalence by 98% within two months compared to untreated cages. However, the emergence of a new generation of cymothoids in treated cages demonstrated that reinfection could occur even in fish exceeding 20 g in weight. These findings emphasise the need for repeated and integrated management strategies aimed at reducing the number of sexually mature females to effectively suppress cymothoid populations over time.

Infestations of parasitic isopods can increase rapidly in aquaculture systems. For example, a natural prevalence of 33% was shown to increase to 98% within 15 months in cage cultures (Sievers et al. 1996). In the same study, the total number of parasitic isopods increased from 309 to 3,987 individuals, while mean infestation intensity rose from 1.4 to 6.1 parasites per fish, illustrating the capacity for rapid population expansion under cultured conditions (Sievers et al. 1996).

The removal of parasitic isopods from culture facilities is challenging, as these parasites often show greater tolerance to chemical treatments than other ectoparasites. Inappropriate treatment concentrations or prolonged exposure can cause significant harm to host fish, requiring careful selection of compounds, dosages, and exposure times. Repeated use of insecticides may also promote parasite resistance, leading to reduced treatment efficacy and the need for higher or longer applications, which can further increase toxicity risks to fish and the surrounding environment.

Manual removal of cymothoid isopods is possible, particularly for larger, externally visible individuals attached to the body surface, buccal cavity, or gill chambers of fish. However, this approach is highly labour-intensive, time-consuming, and impractical at large commercial scales, especially in high-density aquaculture systems. Handling fish individually also increases stress and the risk of secondary infections or injury. Nevertheless, manual removal may still be useful in small-scale operations, broodstock management, or for heavily infected individuals where immediate intervention is required.

In addition to mechanical removal, biological control methods can provide valuable supplementary benefits. Cleaner organisms, such as cleaner fish and shrimp, are well known for removing ectoparasites from host fish in both natural systems and aquaculture environments. These organisms are generally more effective against smaller, mobile isopod stages (e.g. juveniles or temporary ectoparasites such as gnathiids) rather than large, firmly attached cymothoids. For example, Grutter (1999) demonstrated the removal of gnathiid isopods by cleaner fish, while Grutter and Lester (2002) showed that cleaner fish significantly reduced the prevalence and abundance of the temporary corallanid isopod *Argathona macronema* (Bleeker, 1857), particularly over short time scales. Similarly, Bunkley-Williams and Williams (1998) reported that the cleaner shrimp *Ancylomenes pedersoni* was capable of removing juvenile cymothoids from a range of hosts within 24 h. Although their impact on adult cymothoids remains limited, these findings highlight the potential of cleaner species to reduce early-stage infestations and overall parasite loads as part of an integrated pest management strategy.

Several chemical treatments have been evaluated for their effectiveness against cymothoid isopods. Sievers et al. (1995) assessed eight commercial insecticides against *Ceratothoa gaudichaudii* infecting Atlantic salmon (*Salmo salar*). The compounds tested were trichlorfon (Neguvon), dichlorvos (Nuvan 1000), fenthion (Baytex), propoxur (Baygon), fenitrothion (Folithion), azamethiphos (Alfacron 50 WP), cyfluthrin (Solfac), and deltamethrin (K-Othrina EC). Of these, only trichlorfon, dichlorvos, and fenthion were non-toxic to the host fish. Trichlorfon (300 ppm) and dichlorvos (3 ppm) achieved 100% parasite mortality following 60 min of exposure, depending on parasite size, with dichlorvos considered the most effective overall. However, prolonged exposure to dichlorvos was found to pose risks to both fish and the environment.

Deltamethrin has also been shown to be effective against cymothoids. Athanassopoulou et al. (2001) reported that 0.05 mg/L deltamethrin eliminated *C. oestroides* (originally identified as *Anilocra physodes*) on European sea bass (*Dicentrarchus labrax*) within two hours. Similarly, Bouboulis et al. (2004) found that 0.01 mg/L deltamethrin reduced *C. oestroides* prevalence to zero within 24 h, with no parasite recovery after 48 h. Čolak et al. (2019) later demonstrated effectiveness at even lower concentrations (0.0035 mg/L). Trichlorfon has also been recommended at concentrations of 0.5–0.75 ppm for 24 h to control *Alitropus typus* (Chinabut 2002). Caution is required when applying these treatments in warmer waters or on alternative host species, as toxicity may increase and impacts on non-target organisms must be considered.

Organophosphate compounds, including trichlorfon, methyl isoxathion, and dichlorvos, have historically been used against cymothoids (Hatai and Yasumoto 1980, 1982). However, some treatments, such as formalin baths and antimicrobial chemotherapeutants, have shown limited success.

Most ectoparasite treatments rely on chemical baths, which are often stressful, labour-intensive, costly, and site-dependent (Stone et al. 1999). As an alternative, Bouboulis et al. (2004) evaluated medicated feed containing the chitin synthesis inhibitor diflubenzuron (PC90) to treat *C. oestroides* in *D. labrax*. A dosage of 3 mg/kg body weight per day for 14 days resulted in complete parasite removal without adverse effects or reinfection 15 days post-treatment. Feed-based treatments allow for application during winter months and at exposed sites and reduce cross-contamination risks by enabling simultaneous treatment of all cages. However, they readily bind to marine sediments and can persist in the environment for extended periods.

Control of gnathiid isopods presents additional challenges due to their benthic life stages. Freshwater tank washing has been shown to reduce severe infestations (Marino et al. 2004), while Jones and Grutter (2005) successfully used short freshwater baths (3 min) followed by a two-hour praziquantel exposure (0.01 g/L praziquantel dissolved in ethanol on the same day as the fish was collected). Trichlorfon (0.4 ppm) has also been applied in repeated baths to manage gnathiid outbreaks (Hispano et al. 2013). However, chemical treatments often fail to reach benthic adults, allowing populations to recover. Hispano et al. (2013) demonstrated that long-term control of *Gnathia maxillaris* required regular monitoring, precise timing of treatments based on life-cycle knowledge, and a combination of chemical and mechanical removal, including plankton net collection of larvae following chemical exposure.

### Prevention methods

#### Husbandry practices

Preventive strategies are critical in reducing the incidence and severity of isopod infestations in aquaculture systems. Reinfections frequently occur following treatment, emphasising the importance of effective husbandry (mentioned above). Regular removal of dead and moribund fish, replacement of fouled or damaged nets, and positioning cages in areas with stronger currents, lower temperatures, and greater depths have all been shown to reduce parasite burdens (Papapanagiotou et al. 1999). In addition, preventing the introduction of parasites from external sources is essential; minimising the entry of wild or non-cultured fish into farming systems reduces the risk of co-introducing their associated parasite fauna. This can be achieved through the use of exclusion netting, maintaining net integrity, and careful site selection to avoid areas with high densities of wild fish.

As stated previously, some isopods may be introduced by food (Rajkumar et al. 2005b). The use of cultured copepods or filtration of wild zooplankton through fine-mesh nets was recommended to prevent the introduction of infective cymothoid larvae, highlighting the role of feed management in parasite prevention.

Optimal husbandry reduces host stress and susceptibility to infection. Stressors such as handling, overcrowding, and poor water quality can induce immunosuppression, increasing vulnerability to parasitic infections (Papapanagiotou et al. 1999). Additional measures, such as using fine-mesh nets around cages and filtering inflow water in land-based systems, can limit parasite entry (Bragoni et al. 1983, 1984). Separating younger and older fish within cage cultures is particularly important, as ovigerous females on larger fish release infective juveniles that readily infest smaller, more vulnerable hosts (Horton and Okamura 2001). Fallowing of sites and routine parasite monitoring further contribute to long-term control (Čolak et al. 2019).

#### Biological control

Biological control methods offer an alternative to chemical interventions. For species such as *A. typus*, whose juveniles are planktonic, the use of cleaner fish or zooplanktivorous species has been proposed as an effective preventive strategy (Chinabut 2002). Regular freshwater bathing is also widely accepted as a control measure for external marine parasites and may be effective against external cymothoids (Andrews et al. 2013).

Overall, effective prevention and control of parasitic isopods in aquaculture relies on an integrated management approach that combines physical, biological, and husbandry-based strategies, supported by regular monitoring and a thorough understanding of parasite life cycles. Strategies that disrupt parasite transmission at the environmental level are generally more effective than treatments applied after infection, as they reduce the risk of infestation, decrease reliance on chemotherapeutants, and help minimise the development of resistance and negative environmental impacts.

### Future perspectives

Although the past decade has confirmed that cymothoid and related isopods pose a serious and recurring threat to aquaculture production, especially in intensive systems, several critical gaps remain unresolved. Future management of parasitic isopods in aquaculture will require a shift from reactive chemotherapeutic treatments towards preventive, ecosystem-based strategies. Reporting of outbreaks should extend beyond simple descriptions of infection to include comprehensive ecological data on the isopod, thereby supporting future research, improving control strategies, and enabling proactive, ecosystem-based management. Rather than preventing infestation entirely, future interventions may aim to enhance host immune competence, reduce parasite feeding efficiency, or limit pathological effects, thereby improving host survival and growth even in the presence of parasites.

Environmental degradation, declining water quality, and climate change can intensify parasite outbreaks by increasing host susceptibility, as well as enhancing parasite transmission and reproductive rates. Future control of parasitic isopods in aquaculture should prioritise preventive environmental management approaches that disrupt transmission pathways, rather than reliance on post-infection chemotherapeutic treatments. Integrating parasite surveillance with water quality monitoring, improving husbandry practices, and accounting for interactions with wild fish assemblages may enhance early detection and reduce reinfection pressure. In the context of climate change and increasing aquaculture intensity, sustainable control of parasitic isopods will depend on reducing reliance on chemical treatments and implementing long-term environmental management approaches.

Although vaccines are widely used against bacterial and viral diseases in aquaculture (Irshath et al. 2023; Yang et al. 2025), their application against larger ectoparasites such as parasitic isopods remains limited. Nevertheless, growing understanding of host immune responses to ectoparasite feeding and tissue damage suggests that immunisation strategies aimed at reducing parasite survival, feeding success, or pathology may represent a future research direction. Continued exploration of novel biological agents (e.g., bacterial surfactants) and environmental manipulations (filtration, disinfection, UV/ozone treatment) could enhance parasite control without harming fish or humans (Buchmann 2022).

Selective breeding and genetic improvement of cultured fish for increased resistance or tolerance to parasitic infections represents a promising long-term strategy. Even partial resistance, expressed as reduced parasite attachment, slower parasite development, or improved tolerance to tissue damage, could substantially lower infestation impacts and reduce reliance on chemical treatments. Advances in genomic and transcriptomic approaches may allow identification of host genes associated with resistance or tolerance to ectoparasites, enabling marker-assisted selection in breeding programmes (Yang et al. 2025). Such approaches could provide sustainable, non-chemical solutions to parasite management under intensive farming conditions.

Molecular detection of isopods plays a crucial role in aquaculture research by enabling accurate and early identification of parasitic species that may otherwise be difficult to distinguish morphologically. Early detection allows for timely management interventions, reducing the risk of severe infestations and associated economic losses. Furthermore, molecular tools provide insights into parasite diversity, population structure, and transmission pathways, which are essential for developing effective prevention strategies and ensuring the health and sustainability of cultured fish stocks.

New approaches, including natural compounds (plant- and microbial-based approaches), gene editing, immunotherapy, and supportive technologies like nanotechnology and biosensors, offer promising alternatives for sustainable parasite management by limiting drug resistance and environmental harm (Yang et al. 2025). Despite their potential, these approaches are still largely experimental, facing challenges such as variable effectiveness, limited validation under real-world aquaculture conditions, high implementation costs, and uncertainties regarding long-term safety and ecological consequences. Continued research and field trials will be crucial to determine their practicality and optimise their use in aquaculture systems.

Phytotherapy has gained increasing attention as a potential alternative to conventional chemical treatments. Plant-derived extracts and secondary metabolites, including alkaloids, terpenoids, and phenolic compounds, exhibit strong bioactive properties and can function as antimicrobial agents (Effendi et al. 2022). Unlike single-compound chemical drugs, herbal treatments typically consist of multiple active components that act synergistically, disrupting parasite physiology and reducing the likelihood of resistance development. Additionally, plant-based therapies are generally more environmentally friendly, biodegradable, and associated with fewer side effects, while also being cost-effective and widely accessible, particularly in developing regions. Although several plant-derived compounds have shown efficacy against certain parasites, their effectiveness against isopods remains unknown, with some evidence suggesting they may be more suitable for internal parasites. Isopods in general are more difficult to eradicate, and the use of plant- and microbial-based approaches may be less effective than with smaller, internal parasites.

Gene editing (genome manipulation) holds potential for improving disease resistance in fish hosts or for targeting parasite genes involved in processes, such as reproduction and moulting. However, current effects appear to be short-lived or limited to specific life stages, indicating that further research is needed to assess long-term efficacy (Yang et al. 2025). Additionally, the potential ecological consequences of genetically modified organisms entering natural environments remain uncertain and may pose significant risks.

Future studies on parasitic isopods should prioritise detailed experimental investigations of reproductive biology, brood resilience, behaviour, and life-cycle dynamics under varying abiotic and biotic conditions. Species such as *C. oestroides*, which are readily maintained in experimental settings, provide valuable model systems for elucidating key epizootiological processes relevant to cage-reared aquaculture. Information on reproductive cycle length, larval activity, survival in host-free conditions, and sensitivity to mechanical disturbance can be used to identify critical intervention points and optimise the timing of control measures. Such life-cycle-based approaches may allow disruption of transmission pathways and reduce dependence on environmentally harmful chemotherapeutants, which should be reserved for controlled applications only. Ultimately, integrating in vitro and experimental life-cycle studies into parasite management frameworks will be essential for developing ecologically sustainable prevention and control strategies.

## Conclusions

The present study contributes to the growing body of research on parasites in aquaculture by examining cymothoid isopods and other parasitic isopods affecting cultured fish species worldwide. By documenting host–parasite interactions, transmission pathways, and potential management strategies, such as the use of cultured copepods in larval feeding and optimal husbandry, this work addresses the critical need to safeguard the health of production fish. A combination of control measures within an integrated management approach should be used to control infestations, as rotating chemical, medical, biological, and mechanical methods can help slow the emergence of resistance. Furthermore, preventive strategies that focus on environmental management, early detection, and host resilience are increasingly recognised as essential components of sustainable aquaculture.

This review also highlights key gaps in knowledge, particularly regarding parasite life cycles, host specificity, and the ecological dynamics that drive outbreaks under intensive culture conditions. Understanding these factors is crucial for developing more targeted and effective interventions. By synthesising current knowledge and outlining practical and forward-looking strategies, this study provides a foundation for advancing parasite management in aquaculture. It highlights the importance of integrating different approaches to reduce economic losses, enhance fish welfare, and promote sustainable production. Ultimately, these insights support the development of resilient aquaculture systems capable of maintaining high productivity while mitigating the risks posed by parasitic isopods and related ectoparasites.

## Data Availability

No datasets were generated or analysed during the current study.

## References

[CR1] Alvarado V, Schafer JW, Emiquez R, Monras M (1990) Salmonicultura en Chile, estado actual, proyecciones y estado sanitario. Medio Ambiente I 1:9–14

[CR2] Andrews M, Cobcroft JM, Battaglene SC, Valdenegro V, Martin MB, Nowak BF (2013) Parasitic crustaceans infecting cultured striped trumpeter *Latris lineata*. Aquaculture 416–417:280–288

[CR3] Arafat M, Hassan Z, Elmishmishy B, Zaki VH, Zahran E (2026) Ectoparasite prevalence in farmed seabass (*Dicentrarchus labrax*) from Damietta, Egypt: environmental correlations and histological consequences. Vet Res Commun 50(2):166. 10.1007/s11259-026-11084-9 41721917 10.1007/s11259-026-11084-9 PMC12924853

[CR4] Athanassopoulou F, Bouboulis D, Martinsen B (2001) *In vitro* treatments of deltamethrin against the isopod parasite *Anilocra physodes*, a pathogen of sea bass *Dicentrarchus labrax* L. Bull Eur Assoc Fish Pathol 21:26–29

[CR5] Baird Maritime (2025) FAO forecasts rise in 2025 global seafood output driven by aquaculture. https://www.bairdmaritime.com/fishing/aquaculture/fao-forecasts-rise-in-2025-global-seafood-output-driven-by-aquaculture. Accessed 29 Dec 2025

[CR6] Bouboulis D, Athanassopoulou F, Tyrpenou A (2004) Experimental treatments with diflubenzuron and deltamethrin of sea bass, *Dicentrarchus labrax* L., infected with the isopod, *Ceratothoa oestroides*. J Appl Ichthyol 20:314–317. 10.1111/j.1439-0426.2004.00579.x

[CR7] Bragoni G, Romestand B, Trilles J-P (1983) Parasitoses à cymothoadien chez le loup, *Dicentrarchus labrax* (Linnaeus, 1758) en élevage. II. Écophysiologie parasitaire dans le cas de l’étang de Diana (Haute-Corse). Ann Parasitol Hum Comp 58:593–609 6673645

[CR8] Bragoni G, Romestand B, Trilles J-P (1984) Parasitoses à cymothoadien chez le loup, *Dicentrarchus labrax* (Linnaeus, 1758) en élevage. I. Écologie parasitaire dans le cas de l’étang de Diana (Haute-Corse) (Isopoda, Cymothoidae). Crustaceana 47:44–51 6673645

[CR9] Bravo S (1988) Registro de parásitos detectados en salmónidos de cultivo en la X Región. Bol Inf Actual Pfizer, Santiago, Chile

[CR10] Bruce NL (1986) Revision of the isopod crustacean genus *Mothocya* Costa, in Hope, 1851 (Cymothoidae: Flabellifera), parasitic on marine fishes. J Nat Hist 20:1089–1192

[CR11] Buchmann K (2022) Control of parasitic diseases in aquaculture. Parasitology 149:1985–1997. 10.1017/S0031182022001093 35950444 10.1017/S0031182022001093 PMC10090776

[CR12] Bunkley-Williams L, Williams EH Jr. (1998) Ability of Pederson Cleaner Shrimp to remove juveniles of the parasitic cymothoid isopod, *Anilocra haemuli*, from the host. Crustaceana 71:862–869

[CR13] Chinabut S (2002) A case study of isopod infestation in tilapia cage culture in Thailand. In: Arthur JR, Phillips MJ, Subasinghe RP, Reantaso MB, MacRae IH (eds) Primary aquatic animal health care in rural, small-scale aquaculture development. FAO Fish Aquac Tech Pap 406:201–202

[CR14] Čolak S, Kolega M, Mejdandžić D, Župan I, Šarić T, Piplović E, Mustać B (2018) Prevalence and effects of the cymothoid isopod (*Ceratothoa oestroides* Risso, 1816) on cultured meagre (*Argyrosomus regius* Asso, 1801) in the eastern Adriatic Sea. Aquac Res 49:1001–1007. 10.1111/are.13547

[CR15] Čolak S, Barić R, Kolega M, Mejdandžić D, Mustać B, Petani B, Župan I, Šarić T (2019) Effect of the pesticide deltamethrin as a treatment of *Ceratothoa oestroides* infestations of farmed sea bass *Dicentrarchus labrax*. Aquaculture 500:322–326. 10.1016/j.aquaculture.2018.10.044

[CR16] Colorni A, Trilles J-P, Golani D (1997) *Livoneca* sp. (Flabellifera: Cymothoidae), an isopod parasite in the oral and branchial cavities of the Red Sea silverside *Atherinomorus lacunosus* (Perciformes, Atherinidae). Dis Aquat Org 31:65–71

[CR17] de Jesus EC, Cardoso L, Ferreira TH, Martins ML, Rodrigues MDN (2017) *Braga nasuta* (Cymothoidae): an ectoparasite of the giant Amazonian fish *Arapaima gigas* (Osteoglossidae) fingerlings cultured in the Amazon region in Northern Brazil. Acta Sci Biol Sci 39:507–511. 10.4025/actascibiolsci.v39i4.35080

[CR18] Dias MKR, Neves LR, Marinho RGB, Tavares-Dias M (2015) Parasitic infections in tambaqui from eight fish farms in Northern Brazil. Arq Bras Med Vet Zootec 67(4):1070–1076. 10.1590/1678-4162-7592

[CR19] Effendi I, Yoswaty D, Syawal H, Austin B, Lyndon AR, Kurniawan R, Wahyuni S, Al-Harbi A (2022) The use of medicinal herbs in aquaculture industry: a review. In: Chaitanya MVNL (ed) Current aspects in pharmaceutical research and development, vol 7. Book Publisher International, Hooghly, pp 7–20

[CR20] Eissa AE, El Zlitne RA, El-Ramlawy AO, Abu Mhara AA, Alzentani SAA, Abu Leila RHM, Mahmoud AE, Qorany R, Mansour SM, Elbarbary NB, Ibrahim SAA, El Moghazi DF, Prince A, Younis EM, Ragab RH, Attia MM (2025) Role of the cymothoid isopod, *Nerocila bivittata* in spread of *Photobacterium damselae* subsp *piscicida* to Thinlip mullet (*Chelon ramada*). Parasitol Int 109:e103104. 10.1016/j.parint.2025.103104 10.1016/j.parint.2025.103104 40482700

[CR21] Fadel A, Bessat M, Abdel-Aziz M (2020) *Livoneca redmanii* (Isopoda, Cymothoidae) in meagre *Argyrosomus regius*: parasitological and molecular diagnosis and proposed control measure. Dis Aquat Org 140:13–24 10.3354/dao03490 32618284

[CR22] Fogelman RM, Grutter AS (2008) Mancae of the parasitic cymothoid isopod, *Anilocra apogonae*: early life history, host-specificity, and effect on growth and survival of preferred young cardinal fishes. Coral Reefs 27:685–693. 10.1007/s00338-008-0379-2

[CR23] Food and Agriculture Organization (FAO) of the United Nations (2024) The State of World Fisheries and Aquaculture 2024: Global fisheries and aquaculture production reaches a new record high. FAO. https://www.fao.org/newsroom/detail/fao-report-global-fisheries-and-aquaculture-production-reaches-a-new-record-high. Accessed 29 Dec 2025

[CR24] González L, Taylor RS, Bridle AR, Crosbie PBB, Nowak BF (2019) Parasitic isopods *Ceratothoa banksii* (Leach, 1818) and *Nerocila orbignyi* (Guérin-Méneville, 1832) of farmed Atlantic salmon and their potential as vectors of *Neoparamoeba perurans* (Young et al. 2007) in Tasmania. Aquaculture 507:28–34. 10.1016/j.aquaculture.2019.04.008

[CR25] Grutter AS (1999) Cleaner fish really do clean. Nature 398:672–673

[CR26] Grutter AS, Lester RJG (2002) Cleaner fish *Labroides dimidiatus* reduce ‘temporary’ parasitic corallanid isopods on the coral reef fish *Hemigymnus melapterus*. Mar Ecol Prog Ser 234:247–255. 10.3354/meps234247

[CR27] Grutter AS, Morgan JAT, Adlard RD (2000) Characterising parasitic gnathiid isopod species and matching life stages with ribosomal DNA ITS2 sequences. Mar Biol 136:201–205. 10.1007/s002270050677

[CR28] Hadfield KA, Erasmus A, Bruce NL, Smit NJ (2025) Biology and life cycles of parasitic Arthropoda infesting aquatic hosts. In: Smit NJ, Sures B (eds) Aquatic parasitology: ecological and environmental concepts and implications of marine and freshwater parasites. Springer, Cham, pp 125–164. 10.1007/978-3-031-83903-0_6

[CR29] Hatai K, Yasumoto S (1980) A parasitic isopod *Irona melanosticta* isolated from the gill chamber of fingerlings of cultured yellowtail, *Seriola quinqueradiata*. Bull Nagasaki Prefect Inst Fish 6:87–96

[CR30] Hatai K, Yasumoto S (1982) The effects of methyl isoxathion in eliminating the parasitic isopod *Irona melanosticta*. Aquaculture 30:147–150

[CR31] Hispano C, Bultó P, Blanch AR (2013) *Gnathia maxillaris* infestation in exhibition aquaria: control and treatment strategies. J Appl Ichthyol 29:1139–1144. 10.1111/jai.12249

[CR32] Horton T, Okamura B (2001) Cymothoid isopod parasites in aquaculture: a review and case study of a Turkish sea bass (*Dicentrarchus labrax*) and sea bream (*Sparus auratus*) farm. Dis Aquat Org 46:181–187. 10.3354/dao046181 10.3354/dao046181 11710552

[CR33] Horton T, Okamura B (2003) Post-haemorrhagic anaemia in sea bass, *Dicentrarchus labrax* (L.), caused by blood feeding of *Ceratothoa oestroides* (Isopoda: Cymothoidae). J Fish Dis 26:401–406. 10.1046/j.1365-2761.2003.00476.x 12946009 10.1046/j.1365-2761.2003.00476.x

[CR34] Irshath AA, Rajan AP, Vimal S, Prabhakaran VS, Ganesan R (2023) Bacterial pathogenesis in various fish diseases: recent advances and specific challenges in vaccine development. Vaccines 11(2):470. 10.3390/vaccines11020470 36851346 10.3390/vaccines11020470 PMC9968037

[CR35] Jones CM, Grutter AS (2005) Parasitic isopods (*Gnathia* sp.) reduce haematocrit in captive blackeye thicklip (Labridae) on the Great Barrier Reef. J Fish Biol 66:860–864. 10.1111/j.0022-1112.2005.00640.x

[CR36] Lanzing WJR, O’Connor PF (1975) Infestation of luderick (*Girella tricuspidata*) populations with parasitic isopods. Aust J Mar Freshw Res 26:355–361

[CR37] Marino F, Giannetto S, Paradiso ML, Bottari T, de Vico G, Macrì B (2004) Tissue damage and haematophagia due to praniza larvae (Isopoda: Gnathiidae) in some aquarium seawater teleosts. Dis Aquat Org 59:43–47. 10.3354/dao059043 10.3354/dao059043 15212291

[CR38] Mladineo I (2002) Prevalence of *Ceratothoa oestroides* (Risso, 1826), a cymothoid isopode parasite, in cultured sea bass *Dicentrarchus labrax* L. on two farms in middle Adriatic Sea. Acta Adriat 43:97–102

[CR39] Mladineo I (2006) Check list of the parasitofauna in Adriatic Sea cage-reared fish. Acta Vet (Beograd) 56:285–292

[CR40] Nagasawa K, Shirakashi S (2017) *Nerocila phaiopleura*, a cymothoid isopod parasitic on Pacific bluefin tuna, *Thunnus orientalis*, cultured in Japan. Crustacean Res 46:95–101. 10.18353/crustacea.46.0_95

[CR41] Nagasawa K, Shirakashi S, Yamamoto S (2018) *Nerocila japonica* Schioedte & Meinert, 1881 (Isopoda, Cymothoidae) found in a Japanese culture of *Girella leonina* (Richardson, 1846) (Actinopterygii, Kyphosidae). Crustaceana 91:375–377. 10.1163/15685403-00003763

[CR42] Noor El-Deen AE, Zaki MS, Shalaby IS (2018) Some investigations observed in cultured seabass (*Dicentrarchus labrax* L.) infested with *Lernanthropus kroyeri* and *Nerocila orbignyi* and exposed to cadmium pollution during different seasons at Dammaitte Province. In: Kumar V, Kumar M, Prasad R (eds) Phytobiont and Ecosystem Restitution. Springer, Singapore, pp 263–274

[CR43] Nowak BF, Dawson D, Basson L, Deveney M, Powell MD (2004) Gill histopathology of wild marine fish in Tasmania: potential interactions with gill health of cultured Atlantic salmon, *Salmo salar* L. J Fish Dis 27:709–717 15575879 10.1111/j.1365-2761.2004.00593.x

[CR44] Nowak BF, Martin MB, Boltaña S (2020) Parasitic Crustaceans. In: Lovrich G, Thiel M (eds) Fisheries and Aquaculture: Volume 9. Oxford Academic, New York, pp 401–462

[CR45] OECD-FAO (2025) OECD-FAO Agricultural Outlook 2025–2034. 10.1787/601276cd-en. Paris and Rome

[CR46] Ogawa K (2005) Effects in finfish culture. In: Rohde K (ed) Marine parasitology. CSIRO Publishing and CABI Publishing, Collingwood, Australia, pp 378–391

[CR47] Oliveira MSB, Tavares-Dias M (2025) Mutilation on gill filaments of *Piaractus brachypomus* (Characiformes: Serrasalmidae) caused by *Braga patagonica* (Crustacea: Cymothoidae), in the Brazilian Amazon. Rev Bras Parasitol Vet 34(4):e012725. 10.1590/S1984-29612025068 41370556 10.1590/S1984-29612025068 PMC12704772

[CR48] Papapanagiotou EP, Trilles J-P (2001) Cymothoid parasite *Ceratothoa parallela* inflicts great losses on cultured gilthead sea bream *Sparus aurata* in Greece. Dis Aquat Org 45:237–239 10.3354/dao045237 11558733

[CR49] Papapanagiotou EP, Trilles J-P, Photis G (1999) First record of *Emetha audouini*, a cymothoid isopod parasite, from cultured sea bass *Dicentrarchus labrax* in Greece. Dis Aquat Org 38:235–237 10.3354/dao038235 10686675

[CR50] Paperna I, Overstreet RM (1981) Parasites and diseases of mullets (Mugilidae). In: Oren OH (ed) Aquaculture of grey mullets. University Press, Cambridge, pp 411–493

[CR51] Rajkumar M, Kumaraguru Vasagam KP, Perumal P, Trilles J-P (2005a) First record of *Cymothoa indica* (Crustacea, Isopoda, Cymothoidae) infecting the cultured catfish *Mystus gulio* in India. Dis Aquat Org 65:269–272 10.3354/dao065269 16119896

[CR52] Rajkumar M, Perumal P, Trilles J-P (2005b) *Cymothoa indica* (Crustacea, Isopoda, Cymothoidae) parasitizes the cultured larvae of the Asian seabass *Lates calcarifer* under laboratory conditions. Dis Aquat Org 66:87–90 10.3354/dao066087 16175971

[CR53] Rameshkumar G, Ravichandran S, Sivasubramanian K (2013) Secondary microbial infection in carangid fishes due to cymothoid isopod parasites. Natl Acad Sci Lett 36:591–597

[CR54] Sanil NK, Vikas PA, Ratheesh TB, George KC, Vijayan KK (2009) Mortalities caused by the crustacean isopod *Cirolana fluviatilis* in tropical, cage-cultured Asian seabass, *Lates calcarifer*: a case study from the southwest coast of India. Aquac Res 40:1626–1633. 10.1111/j.1365-2109.2009.02263.x

[CR55] Šarušić G (1999) Preliminary report of infestation by isopod *Ceratothoa oestroides* (Risso, 1826), in marine cultured fish. Bull Eur Assoc Fish Pathol 19:110–112

[CR56] Shinn AP, Pratoomyot J, Bron JE, Paladini G, Brooker EE, Brooker AJ (2015) Economic costs of protistan and metazoan parasites to global mariculture. Parasitology 142:196–270. 10.1017/S0031182014001437 25438750 10.1017/S0031182014001437

[CR57] Sievers G, Palacios P, Inostroza R, Dölz H (1995) Evaluation of the toxicity of 8 insecticides in *Salmo salar* and the in vitro effects against the isopode [sic] parasite, *Ceratothoa gaudichaudii*. Aquaculture 134:9–16

[CR58] Sievers G, Lobos C, Inostroza R, Ernst S (1996) The effect of the isopod parasite *Ceratothoa gaudichaudii* on the body weight of farmed *Salmo salar* in southern Chile. Aquaculture 143:1–6

[CR59] Smit NJ, Bruce NL, Hadfield KA (2014) Global diversity of fish parasitic isopod crustaceans of the family Cymothoidae. Int J Parasitol Parasites Wildl 3:188–197. 10.1016/j.ijppaw.2014.03.004 25180163 10.1016/j.ijppaw.2014.03.004 PMC4145142

[CR60] Stepien CA, Brusca RC (1985) Nocturnal attacks on nearshore fishes in southern California by crustacean zooplankton. Mar Ecol Prog Ser 21:91–105

[CR61] Stone J, Sutherland IH, Sommerville CS, Richards RH, Varma KJ (1999) The efficacy of emamectin benzoate as an oral treatment of sea lice, *Lepeophtheirus salmonis* (Kréyer), infestations in Atlantic salmon, *Salmo salar* L. J Fish Dis 22:261–270. 10.1046/j.1365-2761.1999.00176.x

[CR62] Suresh K, Gopi S, Rakesh CG, Ittoop G, Pillai D (2022) Occurrence of infestation with the isopod *Alitropus typus* M. Edwards (Crustacea: Flabellifera: Aegidae) on commercially important freshwater fishes of Kerala, India. J Parasit Dis 46:695–703. 10.1007/s12639-022-01488-0 36091279 10.1007/s12639-022-01488-0 PMC9458801

[CR63] Tavares-Dias M, Araújo CSO, Barros MS, Viana GM (2014) New hosts and distribution records of *Braga patagonica*, a parasite Cymothoidae of fishes from the Amazon. Braz J Aquat Sci Technol 18:91–97

[CR64] Thing CY, Ota Y, Hatai K, Ransangan J (2015) Redescription of *Caecognathia coralliophila* (Monod, 1926) (Crustacea, Isopoda, Gnathiidae), collected from a fish hatchery in Sabah, Borneo Island, Malaysia. Proc Biol Soc Wash 128:51–62

[CR65] Timi JT, Buchmann K (2023) A century of parasitology in fisheries and aquaculture. J Helminthol 97(e4):1–18. 10.1017/S0022149X22000797 10.1017/S0022149X22000797 36631485

[CR66] Trilles J-P, Öktener A (2009) New host records for *Ceratothoa oestroides* and *Anilocra physodes* (Isopoda, Cymothoidae) in Turkish Waters. Kafkas Univ Vet Fak Derg 15:469–471

[CR67] Vagianou S, Athanassopoulou F, Ragias V, Di Cave D, Leontides L, Golomazou E (2006) Prevalence and pathology of ectoparasites of Mediterranean fish, reared under three different environmental and aquaculture conditions in Greece. Isr J Aquac Bamidgeh 58:78–88

[CR68] Valle C, Bayle-Sempere JT, Dempster T, Sanchez-Jerez P, Giménez-Casalduero F (2007) Temporal variability of wild fish assemblages associated with a sea-cage farm in the south-western Mediterranean Sea. Estuar Coast Shelf Sci 72:299–307. 10.1016/j.ecss.2006.10.019

[CR69] Williams EH Jr, Bunkley Williams L (1996) Parasites of offshore big game fishes of Puerto Rico and the Western Atlantic. Puerto Rico Department of Natural and Environmental Resources, San Juan, and the University of Puerto Rico, Mayaguez, Puerto Rico, p 382

[CR70] Williams EH Jr., Bunkley-Williams L (2000) On the generic placement of “*Livoneca* sp.”: a critique of Colorni et al. (1997). Dis Aquat Org 40:233–234 10.3354/dao040233 10843563

[CR71] Yang J, Bhassu S, Rajamanikam A (2025) Charting the future: advanced technologies for sustainable parasite control in aquaculture. Int J Mol Sci 26(21):10738. 10.3390/ijms262110738 41226772 10.3390/ijms262110738 PMC12609390

